# Supplementary material for: Factors affecting genotyping success in giant panda fecal samples
Source: PeerJ. 2017 May 23;5:e3358. doi: 10.7717/peerj.3358 (PMC5444362; doi:10.7717/peerj.3358)
Supplement: Table S1 [file peerj-05-3358-s001.docx]

Supplemental material

Ying ZHU, Hong-Yi LIU, Hai-Qiong YANG, Yu-Dong LI, He-Min ZHANG. 2017. Factors Affecting Genotyping Success in Giant Panda Fecal Samples. PeerJ

Corresponding author: He-Min ZHANG, China Conservation and Research Center for the Giant Panda, No. 98 Tongjiang Road, Dujiangyan, 611800,Sichuan Province, China. Phone: +86-837-6246861; Fax:+86-837-6246776. email address: wolong_zhm@163.com; wolong_zhm@126.com

Table S1 The pairwise comparisons between storage types for each storage time on amplification success.

|  | | 1 month |  | 3 months |  | 6 months |  | All 3 storage times | |
| --- | --- | --- | --- | --- | --- | --- | --- | --- | --- |
| Storage type | | Mean Difference | *P* value | Mean Difference | *P*  value | Mean Difference | *P* value | Mean Difference | *P* value |
| EtOH | EtoH/-20°C | 0.033 | 0.986 | 0.072 | 0.859 | 0.157 | **0.021** | 0.087 | 0.534 |
|  | 2 steps | 0.000 | 1.000 | -0.039 | 0.983 | 0.085 | 0.696 | 0.015 | 0.999 |
|  | DET | 0.020 | 0.998 | 0.033 | 0.991 | 0.209 | **0.017** | 0.087 | 0.534 |
|  | -20°C | 0.052 | 0.927 | 0.360 | **0.000** | 0.529 | **0.000** | 0.314 | **0.000** |
| EtoH | 2 steps | -0.033 | 0.986 | -0.111 | 0.545 | -0.072 | 0.809 | -0.072 | 0.706 |
| /-20°C | DET | -0.013 | 1.000 | -0.039 | 0.983 | 0.052 | 0.931 | 0.000 | 1.000 |
|  | -20°C | 0.020 | 0.998 | 0.288 | **0.001** | 0.373 | **0.000** | 0.227 | **0.001** |
| 2 steps | DET | 0.020 | 0.998 | 0.072 | 0.859 | 0.124 | 0.331 | 0.072 | 0.706 |
|  | -20°C | 0.052 | 0.927 | 0.399 | **0.000** | 0.444 | **0.000** | 0.299 | **0.000** |
| DET | -20°C | 0.033 | 0.986 | 0.327 | **0.000** | 0.320 | **0.000** | 0.227 | **0.001** |
