# Supplementary material for: Factors affecting genotyping success in giant panda fecal samples
Source: PeerJ. 2017 May 23;5:e3358. doi: 10.7717/peerj.3358 (PMC5444362; doi:10.7717/peerj.3358)
Supplement: Table S2 [file peerj-05-3358-s002.docx]

Supplemental material

Ying ZHU, Hong-Yi LIU, Hai-Qiong YANG, Yu-Dong LI, He-Min ZHANG. 2017. Factors Affecting Genotyping Success in Giant Panda Fecal Samples. PeerJ

Corresponding author: He-Min ZHANG, China Conservation and Research Center for the Giant Panda, No. 98 Tongjiang Road, Dujiangyan, 611800,Sichuan Province, China. Phone: +86-837-6246861; Fax:+86-837-6246776. email address: wolong_zhm@163.com; wolong_zhm@126.com

Table S2 The pairwise comparisons between storage time for each storage type on amplification success

|  | Storage time | | Mean Difference | *P* value |
| --- | --- | --- | --- | --- |
| EtoH | 1 month | 3 months | 0.059 | 0.217 |
|  |  | 6 months | 0.052 | 0.088 |
|  | 3 month | 6 months | -0.007 | 0.889 |
| EtoH/-20°C | 1 month | 3 months | 0.098 | **0.009** |
|  |  | 6 months | 0.176 | **0.002** |
|  | 3 month | 6 months | 0.078 | 0.076 |
| 2 steps | 1 month | 3 months | 0.020 | 0.484 |
|  |  | 6 months | 0.137 | 0.051 |
|  | 3 month | 6 months | 0.118 | 0.083 |
| DET | 1 month | 3 months | 0.072 | **0.023** |
|  |  | 6 months | 0.242 | **0.000** |
|  | 3 month | 6 months | 0.170 | **0.000** |
| -20°C | 1 month | 3 months | 0.366 | **0.000** |
|  |  | 6 months | 0.529 | **0.000** |
|  | 3 month | 6 months | 0.163 | **0.003** |
| All storage types | 1 month | 3 months | 0.123 | **0.000** |
|  |  | 6 months | 0.227 | **0.000** |
|  | 3 month | 6 months | 0.105 | **0.000** |
