# Supplementary material for: Factors affecting genotyping success in giant panda fecal samples
Source: PeerJ. 2017 May 23;5:e3358. doi: 10.7717/peerj.3358 (PMC5444362; doi:10.7717/peerj.3358)
Supplement: Table S3 [file peerj-05-3358-s003.docx]

Supplemental material

Ying ZHU, Hong-Yi LIU, Hai-Qiong YANG, Yu-Dong LI, He-Min ZHANG. 2017. Factors Affecting Genotyping Success in Giant Panda Fecal Samples. PeerJ

Corresponding author: He-Min ZHANG, China Conservation and Research Center for the Giant Panda, No. 98 Tongjiang Road, Dujiangyan, 611800,Sichuan Province, China. Phone: +86-837-6246861; Fax:+86-837-6246776. email address: wolong_zhm@163.com; wolong_zhm@126.com

Table S3 The pairwise comparisons between storage types through all storage times on allelic dropout rate

|  | Storage type | Mean Difference | *P* value |
| --- | --- | --- | --- |
| EtOH | EtoH/-20°C | -0.008 | 1.000 |
|  | 2 steps | -0.026 | 0.976 |
|  | DET | -0.040 | 0.898 |
|  | -20°C | -0.193 | **0.001** |
| EtoH | 2 steps | -0.018 | 0.994 |
| /-20°C | DET | -0.032 | 0.951 |
|  | -20°C | -0.185 | **0.001** |
| 2 steps | DET | -0.014 | 0.998 |
|  | -20°C | -0.167 | **0.005** |
| DET | -20°C | -0.153 | **0.011** |
