# Supplementary material for: Factors affecting genotyping success in giant panda fecal samples
Source: PeerJ. 2017 May 23;5:e3358. doi: 10.7717/peerj.3358 (PMC5444362; doi:10.7717/peerj.3358)
Supplement: Table S4 [file peerj-05-3358-s004.docx]

Supplemental material

Ying ZHU, Hong-Yi LIU, Hai-Qiong YANG, Yu-Dong LI, He-Min ZHANG. 2017. Factors Affecting Genotyping Success in Giant Panda Fecal Samples. PeerJ

Corresponding author: He-Min ZHANG, China Conservation and Research Center for the Giant Panda, No. 98 Tongjiang Road, Dujiangyan, 611800,Sichuan Province, China. Phone: +86-837-6246861; Fax:+86-837-6246776. email address: wolong_zhm@163.com; wolong_zhm@126.com

Table S4 The amplification success, allelic dropout and false allele rates from 3 grades fragment length

|  | Amplification success | ADO | FA |
| --- | --- | --- | --- |
| Grade I (<150bp) | 92.9% (2.2%) | 5.6% (2.1%) | 10% (1.6%) |
| Grade II  (150-200bp) | 87.6% (2.2%) | 2.8% (2.1%) | 0 (1.6%) |
| Grade III  (200-300bp) | 92% (1.5%) | 4.7% (1.4%) | 9.6% (1.1%) |

Note: Figures in parentheses meant standard errors. ADO is abbreviation for allele dropout and FA for false allele. The ADO and FA were determined by comparing the genotype amplified from fecal DNA and blood DNA.
