# Supplementary material for: Factors affecting genotyping success in giant panda fecal samples
Source: PeerJ. 2017 May 23;5:e3358. doi: 10.7717/peerj.3358 (PMC5444362; doi:10.7717/peerj.3358)
Supplement: Table S5 [file peerj-05-3358-s005.docx]

Supplemental material

Ying ZHU, Hong-Yi LIU, Hai-Qiong YANG, Yu-Dong LI, He-Min ZHANG. 2017. Factors Affecting Genotyping Success in Giant Panda Fecal Samples. PeerJ

Corresponding author: He-Min ZHANG, China Conservation and Research Center for the Giant Panda, No. 98 Tongjiang Road, Dujiangyan, 611800,Sichuan Province, China. Phone: +86-837-6246861; Fax:+86-837-6246776. email address: wolong_zhm@163.com; wolong_zhm@126.com

Table S5 The pairwise comparisons between repeat motifs on amplification success rate, allelic dropout rate and false allele rate

|  | Repeat motif | | Mean Difference | *P* value |
| --- | --- | --- | --- | --- |
| Amplification success | Di- | Tri- | 0.005 | 0.853 |
|  |  | Tetra- | 0.009 | 0.746 |
|  | Tri- | Tetra- | 0.004 | 0.882 |
| ADO | Di- | Tri- | 0.072 | **0.005** |
|  |  | Tetra- | 0.095 | **0.001** |
|  | Tri- | Tetra- | 0.023 | 0.358 |
| FA | Di- | Tri- | 0.163 | **0.000** |
|  |  | Tetra- | 0.195 | **0.000** |
|  | Tri- | Tetra- | 0.032 | 0.108 |
